# Constructing Controllable Logic Circuits Based on DNAzyme Activity

**DOI:** 10.3390/molecules24224134

**Published:** 2019-11-15

**Authors:** Fengjie Yang, Yuan Liu, Bin Wang, Changjun Zhou, Qiang Zhang

**Affiliations:** 1 Key Laboratory of Advanced Design and Intelligent Computing, Dalian University, Ministry of Education, Dalian 116622, China; yangfengjie5120@163.com; 2 School of Computer Science and Technology, Dalian University of Technology, Dalian 116024, China; liuyuan.dlut@gmail.com; 3 College of Computer Science and Engineering, Dalian Minzu University, Dalian 116600, China

**Keywords:** DNAzyme activity, hairpin DNA, logic circuits, leakage

## Abstract

Recently, DNA molecules have been widely used to construct advanced logic devices due to their unique properties, such as a simple structure and predictable behavior. In fact, there are still many challenges in the process of building logic circuits. Among them, the scalability of the logic circuit and the elimination of the crosstalk of the cascade circuit have become the focus of research. Inspired by biological allosteric regulation, we developed a controllable molecular logic circuit strategy based on the activity of DNAzyme. The E6 DNAzyme sequence was temporarily blocked by hairpin DNA and activated under appropriate input trigger conditions. Using a substrate with ribonucleobase (rA) modification as the detection strand, a series of binary basic logic gates (YES, AND, and INHIBIT) were implemented on the computational component platform. At the same time, we demonstrate a parallel demultiplexer and two multi-level cascade circuits (YES-YES and YES-Three input AND (YES-TAND)). In addition, the leakage of the cascade process was reduced by exploring factors such as concentration and DNA structure. The proposed DNAzyme activity regulation strategy provides great potential for the expansion of logic circuits in the future.

## 1. Introduction

The specificity and predictability of Watson-Crick base pairing make DNA an ideal material for nanoengineering. So far, DNA nanotechnology has been widely used to construct DNA logic operations [1,2,3,4], molecular switches [5,6], catalytic amplifiers [7,8,9], cyclic networks [10,11], biochemistry reaction network [12,13,14], DNA coding [15,16], and digital image processing [17,18,19]. In particular, DNA logic circuits have become a research hotspot due to their programmability and scalability advantages. Related research fields include sensors [20,21,22,23], gene regulation [24,25], disease monitoring [26], nanorobots [27,28] and other areas.

DNA strand displacement [29,30] is a dynamic DNA nanotechnology, which is dynamically regulated through covalent connections between the toehold domain and branch migration domain. To maximize the dynamic adjustment of DNA strand displacement, a variety of strategies have been devised. However, in large regulatory systems with various dynamic interactions, a method may be needed in combination with DNA strand displacement to achieve more complex molecular logic calculation operations. At present, DNA strand displacement has been combined with many bioengineering methods, such as DNA self-assembly [31,32], restriction enzymes [33,34,35], and DNAzyme [36,37,38,39].

Interestingly, DNA strand displacement can be used as a regulator of DNAzyme structure to develop complex molecular logic circuits. Due to the high specific recognition of DNAzyme, it has outstanding applications in the construction of the molecular logic gate [40,41,42,43], half-adder, half-subtractor, multiplexer [44,45,46,47], molecular automaton [48], and cascade circuit [49,50,51]. This method of DNAzyme involvement in construction can regulate DNA structure through specific sequence allostericity without constructing multiple strand interactions to modulate the DNA sequence. The high specificity of DNAzyme catalyzes the cleavage of ribophosphodiester linkage, and the resulting DNA strand acts as a trigger for downstream reactions. Therefore, to achieve dynamic linkage between adjacent modules, adjusting the activity of the DNAzyme is a critical step in logical computation. To regulate the activity of DNAzyme, various methods have been applied. For example, researchers have explored adjusting the metal ion concentration [52,53,54], temperature [55,56], and pH value [57,58], in addition to controlling the activity of DNAzyme through a secondary structure [59,60]. However, in the multi-layer connected circuit, strand-to-strand crosstalk problems often occur between the upstream circuit and the downstream circuit, causing leakage. In systems with high leakage, the total leakage contributes greatly to the logic circuit signal and directly affects the performance of the system. In particular, the response time of cascade circuits is generally longer than that of a single logic gate. Therefore, it is very important to develop a leak control mechanism under prolonged reaction conditions.

Here, we developed a strategy for controllable logic circuits based on Mg^2+^-dependent E6 DNAzyme activity. This strategy uses a complete hairpin structure sequence as a template, and the DNA hairpin consists of a stem loop that is highly stable when a certain number of base pairs are matched. The key of the experiment is to lock a part of the DNAzyme-conserved domain and a recognition arm into the DNA hairpin structure, and to induce the DNAzyme to switch from the inactive state to the active state by adding the input strand. We first constructed a series of logic gates such as YES gate, AND gate and INHIBIT gate. Then, to solve the computational complexity, a demultiplexer logic circuit with the ability to perform multi-component functions was implemented on a single logic gate basis. At the same time, to verify the scalability of the experiment, two layers of YES-YES and YES-TAND cascade circuits were constructed. Finally, verification from the analysis of concentration and DNA structure solved the leakage problem in the cascade circuit. Furthermore, the logic system has the advantage that these logic circuits are implemented on an isothermal and protein-free platform. Except for the hairpin structure, which needs to be double-helical after polymerase chain reaction (PCR) annealing, all reactions need to be completed in one step and are easy to use. We proved the feasibilities of regulating DNA logic circuits. The cascade of DNA strand displacement and DNAzyme collaboration enables dynamic linking between adjacent modules, allowing researchers to have more possibilities in building nanoscale complex logic circuits.

## 2. Results and Discussion

### 2.1. YES Gate

**Figure 1:**
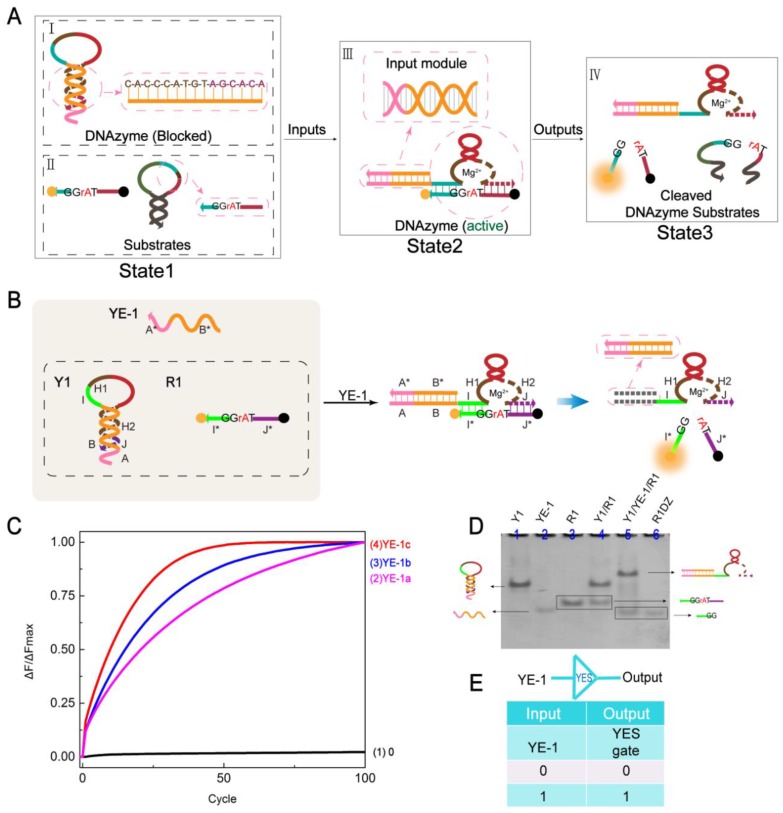
(**A**) Module design of controllable logic gate based on DNAzyme activity. Arrows represent 5′–3′. (**B**) Schematic of the YES gate. The X and X* domains of the entire paper represent complementary base regions. H1 and H2 are the catalytic core components of Mg^2+^. (**C**) Time dependence normalized fluorescence intensity changes according to the input sequence differences (ΔF/ΔFMax). Curve 1 indicates that no input exists and curves 2–4 represent a difference in the sequence of the input strand. The time interval is 3 min, 100 cycles. All data represent the average of three replicates. (**D**) Gel analysis of the YES gate reaction using 12% PAGE. Lane 1: Y1; Lane 2: YE-1; Lane 3: R1; Lane 4: Y1 and R2, representing no input; Lane 5: Y1, YE-1, and R1; Lane 6: R1DZ. Note that because the R1 sequence is too short to appear easily on the PAGE diagram, the base T is added to extend the sequence without affecting the experimental results. (**E**) YES gate logic symbol and truth table. The whole paper 1 represents the input strand, and 0 represents no input strand.

As shown in Figure 1A, State1 has two parts (black dotted boxes I and II). I is a DNA hairpin structure comprising a DNAzyme sequence, and the sequence of the DNAzyme protected by the stem is shown in a pink rounded rectangular dotted line. The DNAzyme is in a locked state. II is the two substrate strands corresponding to the DNAzyme, and the two forms of the substrate (single-strand and small hairpin structures) are shown. Under the action of appropriate input and Mg^2+^, through the strand displacement reaction, III forms two modules: Input module (pink rounded rectangular dotted frame in III) and DNAzyme (active) (pink circular dotted frame in III). State1 is converted to State2. The DNAzyme is paired with a base of the substrate to cause a cleavage site (TrAGG) to be recognized to produce a signal. Eventually State2 is transformed into State3. IV is the final form of the substrate. To demonstrate the feasibility of DNAzyme activity regulation strategies, the basic YES gate was constructed as described below.

The structure of the YES gate is shown in Figure 1B. The system consists of a hairpin structure Y1, a ribonuclease (rA)-containing DNA substrate R1 (the fluorophore/quencher labeled substrate, 5′-FAM, 3′-BHQ1), and an input strand YE-1 (arrows represent 5′–3′). The hairpin structure Y1 and the substrate strand R1 initially coexist in the solution and do not react. By adding the input strand YE-1, the toehold domain A of Y1 is first combined with the A* of YE-1, and then by branch migration, the stem of the hairpin is completely opened. The resulting stable complex exposes the base of the DNAzyme, and the activated DNAzyme cleaves the substrate R1 at the cleavage site (TrAGG), the fluorescence-quench group is separated and the signal is generated. Figure 1E shows the logical symbol and truth table of the YES gate.

First, the YES gate was determined by fluorescence detection. As shown in Figure 1C, the fluorescence signal added to the input strand was significantly increased (curves 2–4). Conversely, curve 1 had no input strand involvement and no apparent fluorescent signal was observed. This demonstrates that the YES gate was successful. Furthermore, curves 2–4 are a comparison of the input strand YE-1. Being concerned that the rigidity of the Input module would affect the bending of the DNAzyme, the number of pairs of B and B* was explored. YE-1c was the optimal input of YES gate. In addition, to further verify the hypothesis, the number of base ‘T’ at the “stem-loop” junction of the hairpin was compared (Figure S1).

Then, the YES gate was confirmed by native polyacrylamide gel electrophoresis (PAGE). As shown in Figure 1D, lane 4 was not added to the input strand, so that the two bands Y1 and R1 can be clearly seen. In the presence of YE-1, a new band (complex Y1/YE-1) and a cleaved substrate (lane 5) were produced in the solution [Y1]:[YE-1]:[R1] = 1:1.2:1.5.

At the same time, to better verify the fluorescence signal response, a control experiment with different input concentrations (Figure S2) was designed with the concentration of other components in the solution unchanged.

### 2.2. AND Gate

**Figure 2:**
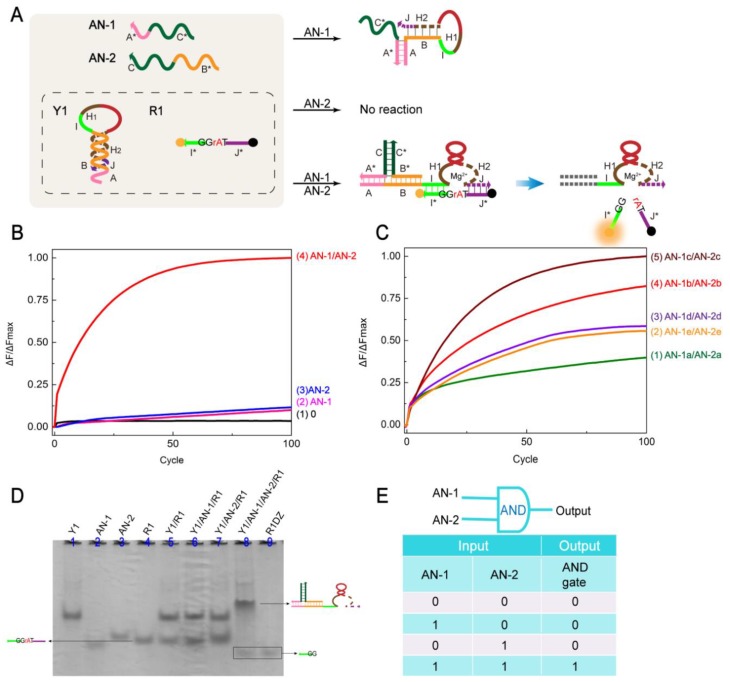
(**A**) Schematic of the AND logic gate. (**B**) Time-dependent normalized fluorescence intensity change (ΔF/ΔFMax) at different inputs. Curves 1–4 reflect the change in fluorescence of the AND gate at different inputs. The time interval is 3 min, 100 cycles. All data represent the average of three replicates. (**C**) Time-dependent normalized fluorescence intensity varies with input sequence differences (ΔF/ΔFMax). Curves 1–5 indicate that AN-1 and Y1 B domain have 0 bp (AN-1a), 1 bp (AN-1b), 2 bp (AN-1c), 3 bp (AN-1d), 4 bp (AN-1e) paired fluorescence changes. AN-2 varies with the change of AN-1. The time interval is 3 min, 100 cycles. All data represent the average of three replicates. (**D**) Gel analysis of the AND gate reaction using 12% PAGE. Lane 1: Y1; Lane 2: AN-1; Lane 3: AN-2; Lane 4: R1; Lane 5: Y1 and R1; Lane 6: Y1, AN-1 and R1; Lane 7: Y1, AN-2 and R1; Lane 8: Y1, AN-1, AN-2, and R1; Lane 9: R1DZ. (**E**) AND gate logic symbol and truth table.

To further explore the possibility of dual-input regulation of DNAzyme activity, an AND gate based on the input strand, DNA hairpin, and substrate cooperation was constructed (Figure 2A). To ensure the uniformity of the experiment, the AND gate uses the same hairpin and substrate (Y1 and R1) as the YES gate. In the AND gate system, when only AN-1 is added, Y1 cannot be fully opened. The structure of the DNAzyme cannot be released (and because of the weak binding strength of AN-1 and Y1, it easily falls off). The final DNAzyme was not activated and no signal was produced. Similarly, when there is only input AN-2, no exposed base of Y1 binds to AN-2, the strand in solution does not react, and no signal was produced. However, When AN-1 and AN-2 are simultaneously added, AN-1, AN-2, and Y1 can form a stable three-way structure. AN-1 is in close proximity to AN-2 and then the hairpin is opened. The DNAzyme in Y1 is exposed and cleaved at the recognition site (TrAGG) with ribonucleobase (rA). There is a signal output that produces an AND gate. Therefore, two inputs are required to activate the DNAzyme, and when there is only one input, the DNAzyme cannot be released and remains inactive. The logical symbol and truth table of the AND gate are shown in Figure 2E.

Fluorescence detection and gel electrophoresis were used to analyze the response of the AND gate (Figure 2B,D). In fluorescence detection, both input strands coexist (AN-1 and AN-2), and the fluorescence signal is significantly enhanced (Figure 2B, curve 4). There was no significant increase in fluorescence intensity with no input or only one input (Figure 2B, curves 1–3). As shown in Figure 2D, the addition of AN-1 and AN-2 inputs has a new band generation (lane 8), and the newly generated band corresponds to lane 9. When no input or only one input is present, no product is produced and the Y1 and R1 bands remain intact (lanes 5, 6, 7) [Y1]:[AN-1]:[AN-2]:[R1] = 1:1.2:1.2:1.5.

To find the best AND gate input and increase the reaction rate, we optimized the input strand. The complementary number of Y1 B domain and AN-1 was processed, that is, AN-1 and Y1 have 0 bp, 1 bp, 2 bp, 3 bp, 4 bp complementarity, and the corresponding input strand AN-2 was modified accordingly (specific sequence reference Table S1). As shown in Figure 2C, curves 1–5 reflect the increase in fluorescence intensity at different input strand sequences. In this experiment, the substrate concentration remained constant and the concentration ratio of all strands in each system was 1:1. By observing these fluorescence curves, we found that when the AN-1c and AN-2c input strands were added, the amount of cut was the largest. The increase was curve 1 < curve 2 < curve 3 < curve 4 < curve 5 (AN-1a/AN-2a < AN-1e/AN-2e < AN-1d/AN-2d < AN-1b/AN-2b < AN-1c/AN-2c). Therefore, the input strands AN-1c and AN-2c are the optimal inputs for the AND gate.

At the same time, to better verify the fluorescence signal response, a control experiment with different input concentrations (Figure S3) was designed with the concentration of other components in the solution unchanged.

In addition, the regulation of DNAzyme activity by three inputs was explored. The schematic of TAND is depicted in Figure S4. The gel electropherogram and fluorescence profile of TAND are given in Figures S5 and S6.

### 2.3. INHIBIT Gate and Demultiplexer

**Figure 3:**
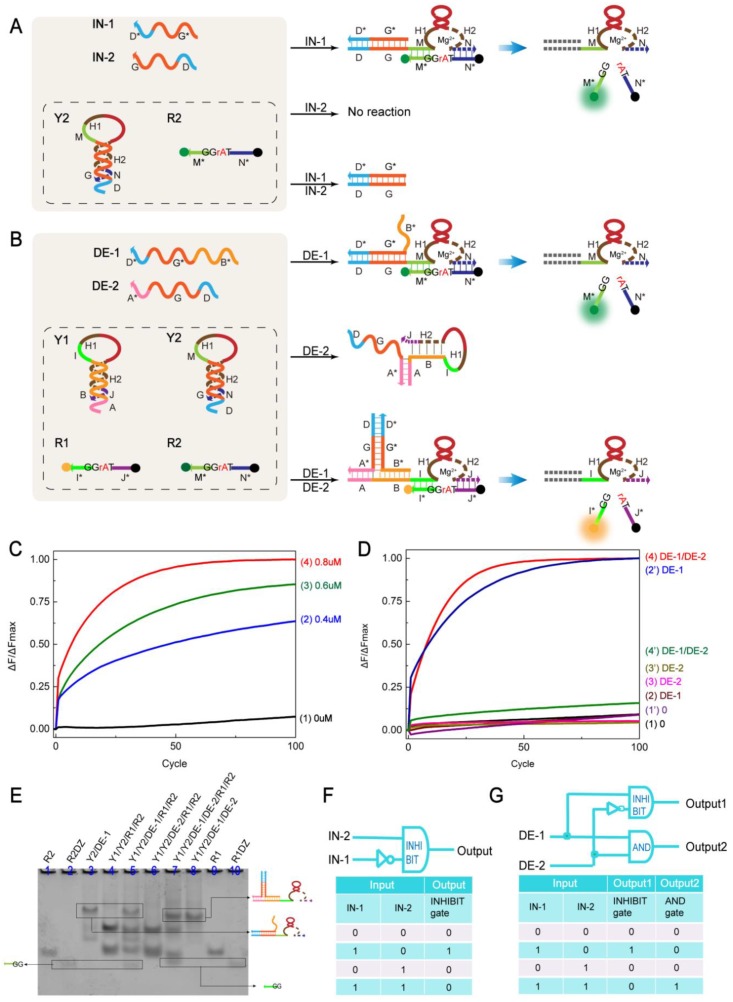
(**A**) Schematic of the INHIBIT logic gate. (**B**) Schematic diagram of the demultiplexer logic circuit. (**C**) Time-dependent normalized fluorescence intensity changes (ΔF/ΔFMax) at different input concentrations. Curves 1, 2, 3, and 4 reflect the fluorescence changes of input strand IN-1 at 0 µM, 0.4 µM, 0.6 µM, and 0.8 µM, respectively. The other strand concentrations in the solution were maintained at 0.3µM. The sampling interval is 3 min, 100 cycles. All data represent the average of three replicates. (**D**) Time-dependent normalized fluorescence intensity change (ΔF/ΔFMax) at different inputs. Curves 1–4 reflect the fluorescence change of the AND gate of the demultiplexer logic circuit at different inputs. Curve 1′–4′ reflect the fluorescence change of the INHIBIT gate of the demultiplexer logic circuit at different inputs. The sampling interval is 3 min, 100 cycles. All data represent the average of three replicates. (**E**) Gel analysis of the YES gate reaction using 12% PAGE. Lane 1: R2; Lane 2: R2DZ; Lane 3: Y2 and DE-1; Lane 4: Y1, Y2, R1, and R2; Lane 5: Y1, Y2, DE-1, R1, and R2; Lane 6: Y1, Y2, DE-2, R1, and R2; Lane 7: Y1, Y2, DE-1, DE-2, R1, and R2; Lane 8: Y1, DE-1, and DE-2; Lane 9: R1; Lane 10: R1DZ. Note that since the R1 and R2 sequences are too short to appear easily on the PAGE diagram, the base T is added to extend the sequence without affecting the experimental results. (**F**) INHIBIT gate logic symbol and truth table. (**G**) Demultiplexer logic circuit symbol and truth table.

Figure 3A depicts the organization of the INHIBIT gate. The mechanism consists of the hairpin structure Y2, a ribonuclease (rA)-containing DNA substrate R2 (the fluorophore/quencher labeled substrate, 5′-HEX, 3′-BHQ1) and the input strands IN1, IN2. IN1 can bind to Y2 to form a stable complex that can activate DNAzyme. The activated DNAzyme cleaves the substrate R2 at the cleavage site (TrAGG) and the fluorescence intensity increases. However, when both IN1 and IN2 are added, the preferential assembly of IN1 and IN2 results in the vast majority of DNAzyme content in Y2 remaining in an inactive state. Only a small number of signals are generated. In addition, there is no signal output without input or only IN2 input. Figure 3F shows the logical symbol and truth table of INHIBIT.

To verify the effect of the concentration of the input strand IN-1 on the INHIBIT logic gate, real-time monitoring was performed using fluorescence (Figure 3C). Curves 1–4 represent the change in fluorescence intensity at input IN-1 concentrations of 0 µM, 0.4 µM, 0.6 µM, and 0.8 µM, respectively. This proves the success of the INHIBIT logic gate. In addition, gel electropherograms and fluorescence plots with different inputs are given in Figure S7.

To fully demonstrate the parallel working ability of biomolecules and improve the working efficiency of molecular devices, it was necessary to construct more complex dynamic control molecular logic circuits. Complex logic circuits require at least two logic gates to be integrated with multiple inputs and outputs. Through the design of the input strand, the AND gate and the INHIBIT gate are combined to construct the demultiplexer logic circuit (Figure 3B). The demultiplexer is composed of a hairpin structure Y1, Y2, ribonuclease (rA) modified substrate strand R1, R2 (R1: 5′-FAM, 3′-BHQ1, R2: 5′-HEX, 3′-BHQ1), and the input strand DE-1, DE-2. To ensure compatibility with a single logic gate, the hairpin structure (Y1, Y2) and the substrate (R1, R2) still followed the sequence of logic gates. In the presence of DE-1, the DNAzyme in Y2 is released; when both DE-1 and DE-2 are present, the DNAzyme in Y1 is released. The DNAzyme in Y1, Y2 recognized the respective substrate binding arms for cleavage, producing a signal output. Conversely, in the absence of input or only DE-2 as an input, no signal is generated. Figure 3G shows a logical symbol and truth table for the demultiplexer.

The fluorescence experiment of the demultiplexer logic circuit is shown in Figure 3D. Curves 2′ and 4 correspond to the case where input strands DE-1, DE-1, and DE-2 were added, respectively, and significant fluorescence increments were observed. Curve 4′ had only a slight increase in fluorescence. No significant fluorescence formation was observed in the other curves. This result demonstrates the success of the demultiplexer logic circuit.

The gel electrophoresis experiment is shown in Figure 3E. In lanes 4 and 6, both R1 and R2 were not consumed, indicating that there was no reaction in the solution without input or only DE-2. The band produced by lane 5 corresponds to the band of lane 2, indicating that substrate R2 is cleaved in the presence of input DE-1. The frequency band generated by lane 7 corresponds to the frequency b and of lane 10, indicating that substrate R1 is cleaved when both DE-1 and DE-2 are present. [Y1]:[Y2]:[DE-1]:[DE-2]:[R1]:[R2] = 1:1:1.2:1.2:1.5:1.5. This result is consistent with the truth table of the demultiplexer logic circuit.

### 2.4. YES-YES Cascading Logic Circuit

**Figure 4:**
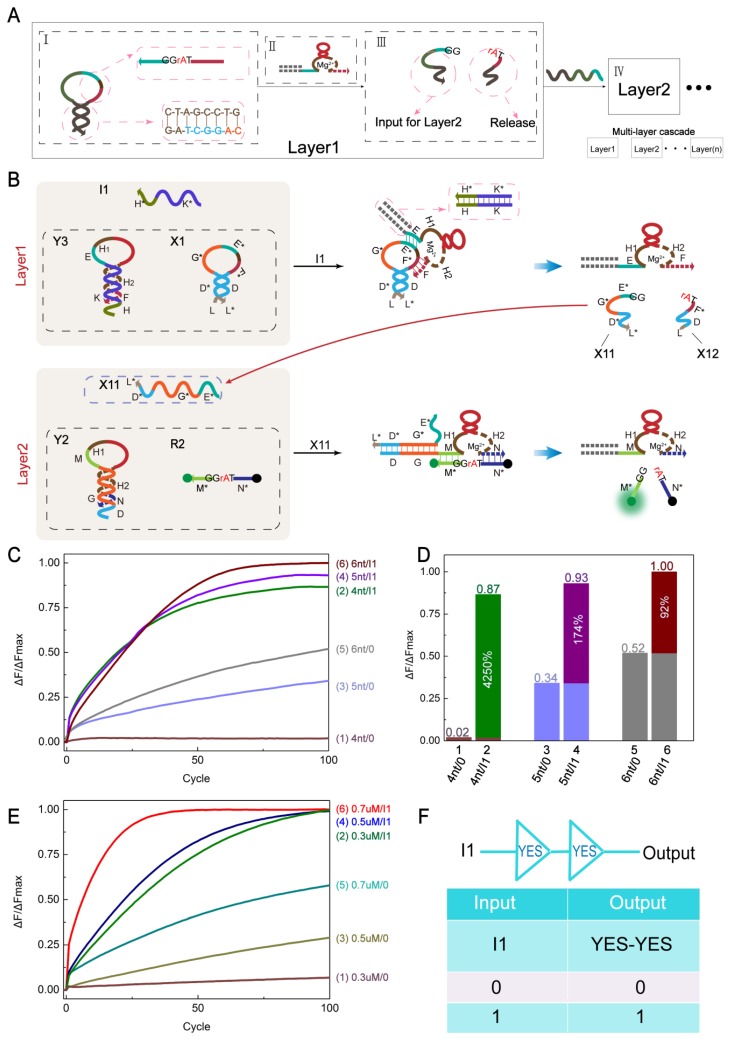
(**A**) Module design of controllable cascading logic circuit based on DNAzyme activity. (**B**) Schematic of the YES-YES logic circuit. (**C**) Time-dependent normalized fluorescence intensity change (ΔF/ΔFMax) when the number of bases in the D domain of Y2 is different. Curves 1, 3, and 5 are changes in fluorescence intensity at no I1, with D domains of 4 nt, 5 nt, and 6 nt, respectively. Curves 2, 4, and 6 are the changes in fluorescence intensity in the presence of I1, corresponding to curves 1, 3, and 5, respectively. The sampling interval is 6 min, 100 cycles. All data represent the average of three replicates. (**D**) Statistical analysis of the number of bases of the D domain of Y2 in Layer2 (corresponding to Figure C). The percentage of relative fluorescence increase ((ΔF(1) − ΔF(0))/ΔF(0)%) is indicated in columns 2, 4, and 6. The reaction time is 10 h. (**E**) Time-dependent normalized fluorescence intensity change (ΔF/ΔFMax) at different concentrations. Curves 1, 3, and 5 are fluorescence intensities without I1, with system concentrations of 0.3 µM, 0.5 µM, and 0.7 µM, respectively. Curves 2, 4, and 6 are changes in fluorescence intensity in the presence of I1, corresponding to curves 1, 3, and 5, respectively. The substrate R2 concentration was fixed at 0.3 µM. The sampling interval is 6 min, 100 cycles. All data represent the average of three replicates. (**F**) YES-YES logic circuit symbol and truth table.

Challenges in DNA computing include implementing series connection of different modules and eliminating crosstalk generated therein. To test the scalability of this strategy-regulated cascaded logic circuit, we designed a small hairpin structure with ribonucleobase (rA) modification (Figure 4A). Layer1 consists of three parts. I is a concrete display of the structure of the small hairpin. The pink circular dotted frame on the ring is the binding arm of the DNAzyme. The stem is designed to prevent crosstalk from downstream logic circuits. II indicates a composite capable of opening a small hairpin. As the DNAzyme is involved in II, the loop of the small hairpin is cleaved. The complementary base at the stem is insufficient to support the structure and is cleaved into two strands, which are shown in III. One of them acts as the input strand for Layer2 and continues to participate in the next level of reaction. Therefore, the cascade of multilayer logic circuit is realized.

A two-layer cascaded YES-YES gate was created (Figure 4B). The cascade circuit is composed of two parts, Layer1 and Layer2. Layer1 consists of a hairpin structure Y3, a small hairpin structure X1 modified by ribonucleobase (rA) and an input strand I1. I1 is the input to the Layer1 YES gate. After the addition of I1, the hairpin structure Y3 is opened, the DNAzyme in Y3 is activated, and then X1 is cleaved at the cleavage site (TrAGG). The resulting strand X11 acts as an input to Layer2. The hairpin structure Y2 and the substrate R2 (5′-HEX, 3′-BHQ1) coexist but do not react in Layer2. However, with the participation of X11, the YES gate of Layer2 is completed, producing an output signal. Figure 4F shows a logical symbol and truth table for the YES-YES logic circuit.

For proper operation of the system, the following conditions must be met: (1) Y3 and X1 do not react in solution when there is no input, (2) DNAzyme released after strand displacement of I1 and Y3 are able to cleave X1, and (3) the structure of the small hairpin X1 needs to be specially designed. The stem region must ensure that it does not react with the downstream without input and that it can be automatically separated after being cracked. Therefore, the length of the Y2 toehold and the concentration of the reactants play a major role in the optimization of the system. To verify these hypotheses, we explored both the length of the Y2 toehold and the concentration of the reactants.

Figure 4C is a graph showing the fluorescence change of the system when the length of the toehold domain (D domain) of Y2 is different. The case where the toehold domain is 4 nt, 5 nt, and 6 nt was explored (the L domain of X1 has 2 nt, 1 nt, and 0 nt). Curves 1, 3, and 5 had different rises when there was no I1. The increase was 4 nt < 5 nt < 6 nt (Y2a < Y2b < Y2). Curves 2, 4, and 6 had a significant increase in fluorescence intensity with the participation of I1. [Y3]:[I1]:[X1]:[Y2a]:[R2] = 1:1.2:1:1:1 (The same applies to Y2b andY2.). A statistical analysis of the comparison of the toehold field of Y2 is given in Figure 4D. Comparing the effects of the leakage of columns 1, 3, and 5, the fluorescence intensities of columns 2, 4, and 6 increased by 4250%, 171%, and 92%, respectively. Therefore, we obtained the best experimental results when the toehold domain of the reaction strand Y2 was 4 nt (the L domain of X1 is 2 nt).

Next, we explored the effect of the concentration of the reactants on the performance of the system. The change in the fluorescence curve is reflected in Figure 4E. Among them, the substrate R2 concentration was always maintained at 0.3 µM. Curves 1, 3, and 5 were observed to have different rises on the fluorescence curve in the absence of I1, with a fluorescence rise of 0.3 µM < 0.5 µM < 0.7 µM. The fluorescence intensity of curves 2, 4, and 6 increased significantly with the participation of input strand I1. It was observed that although the reaction rate was significantly increased at a high concentration, the corresponding leakage was also increased. A statistical analysis of the concentration comparison is given in Figure S8B. Comparative analysis showed that the system concentration of 0.3 µM gives the best performance.

In addition, we used PAGE analysis to verify the YES-YES logic circuit (Figure S9), which also proved the success of the YES-YES logic circuit.

### 2.5. YES-TAND Cascading Logic Circuit

**Figure 5:**
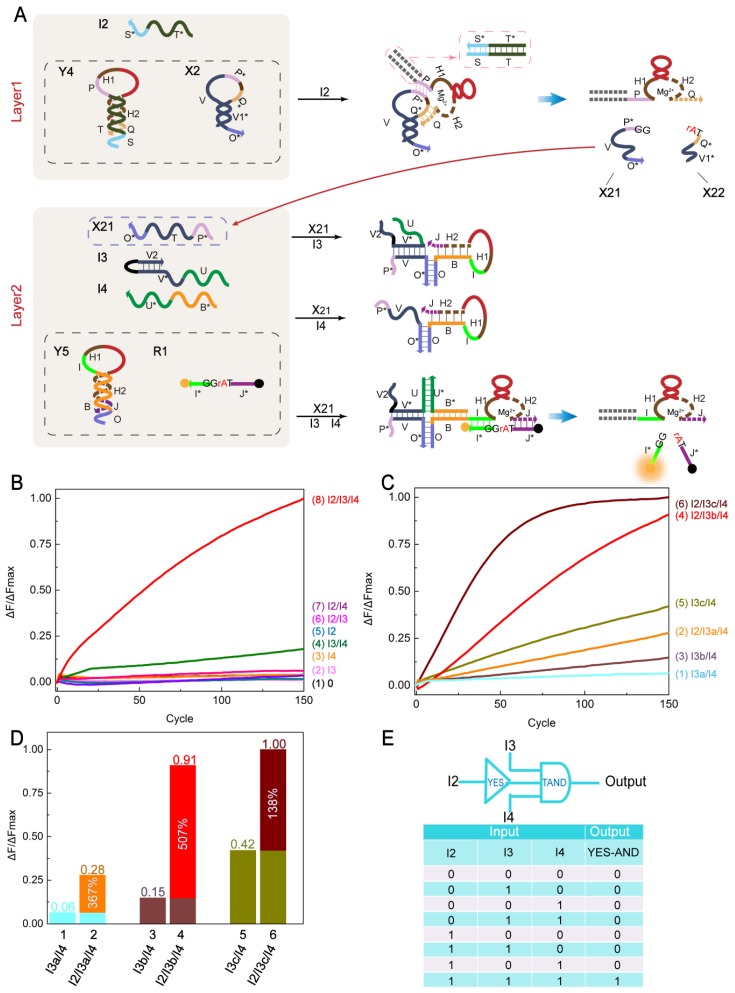
(**A**) Schematic of the YES-TAND logic circuit. Layer2 only shows the reaction with Y5. (**B**) Time-dependent normalized fluorescence intensity change (ΔF/ΔFMax) at different inputs. Curves 1–8 reflect the change in fluorescence of YES-TAND at different inputs. The sampling interval is 6 min, 100 cycles. All data represent the average of three replicates. (**C**) Time-dependent normalized fluorescence intensity change (ΔF/ΔFMax) when the input strand I3 sequence is different. Curves 1, 3 and 5 reflect changes in fluorescence in the absence of I2, with the V and V* domains of I3 having 8 bp, 7 bp and 0 bp complementarity, respectively. Curves 2, 4, and 6 reflect the change in fluorescence in the presence of I2, corresponding to curves 1, 3, and 5, respectively. I4 was present in all tubes of this experiment. The sampling interval is 6 min, 100 cycles. All data represent the average of three replicates. (**D**) Statistical analysis of the number of bases in the V2 region of I3 in Layer2 (corresponding to Figure C). Comparing the leaks of columns 1, 3, and 5, the percentage of relative fluorescence increase is indicated in columns 2, 4, and 6 ((ΔF(1) – ΔF(0))/ΔF(0)%), respectively. The response time is 15 h. (**E**) YES-TAND logic circuit logic symbol and truth table.

Next, we explored the logic cascade circuit of YES-TAND (Figure 5A). The YES-TAND logic circuit consists of Layer1 and Layer2. Layer1 consists of hairpin structure Y4, ribonucleobase (rA) modified small hairpin structure X2, and input strand I2. Upon the addition of I2, the stable duplex formed by Y4 and I2 is sufficient to release the DNAzyme in Y4. The DNAzyme is then base paired with X2, the cleavage is performed after recognition (TrAGG), and then X21 is released as the input toLayer2. Layer2 consists of 5 strands: hairpin structure Y5, substrate R1 (5′-FAM, 3′-BHQ1), input strands I3 and I4, and output strand X21 generated by the upstream reaction. In Layer2, the hairpin Y5 cannot be opened when I3 and X21 are added, so the blocked DNAzyme cannot be released and no output signal is produced. The hairpin Y5 cannot be opened when I4 and X21 are added. On the one hand, since I4 does not have a base complementary to the V domain of x21, a stable structure cannot be formed. On the other hand, Y5 also has no naked base bound to I4. So no output signal is generated. Only when I3 and I4 are simultaneously added and cooperate with X21 and Y5 can it form a stable four-way structure. When the three input strands are in close proximity, the DNAzyme is released, producing an output signal. Figure 5E shows a logical symbol and truth table for the YES-TAND logic circuit.

The YES-TAND logic circuit is analyzed by fluorescence detection (Figure 5B). Curve 8 is the case where all three input strands I2, I3 and I4 are present and a significant fluorescent signal output is observed. Curve 4 is the case where I3 and I4 are present, and fluorescence signal generation that is higher than other background signals is observed. Curves 1–3 and 5–7 are cases other than the above two, in which no significant fluorescence signal generation was observed. [Y4]:[I2]:[X2]:[Y5]:[I3b]:[I4]:[R1] = 1:1.2:1:1:1:1:0.7.

The small hairpin structure X2 differs from X1 in that: (1) small hairpin X2 has a toehold field combined with Y5 and (2) the base protected at the stem is designed to prevent assembly with I3 to further prevent crosstalk with Layer2. Since X21 and I3 have 12 bp complementarity and X2 only protects 8 nt, there is still a risk of leakage, so we also created complementary pairs for I3. This hypothesis is also confirmed by the comparison of several sets of fluorescence curves in Figure 5C. It can be seen from curves 1, 3 and 5 that when I2 is absent, the fluorescence curves rise to different degrees, and the ascending contrast is 8 bp < 7 bp < 0 bp (I3c < I3b < I3a). Curves 2, 4, and 6 reflect the change in the fluorescence intensity of I2. While only a slight increase in fluorescence was observed for curve 2, significant increases in fluorescence signals were observed for curves 4 and 6. It can be concluded that as the I3 complementary logarithm increases, the background signal becomes increasingly negligible. However, the corresponding dominant fluorescence curve rises more and more slowly. [Y4]:[I2]:[X2]:[Y5]:[I3b]:[I4]:[R1] = 1:1.2:1:1:1:1:0.7 (The same applies to I3a and I3c.). We performed a statistical analysis of the three sets of I3 experiments (Figure 5D). Comparing the leakage of columns 1, 3, and 5, the fluorescence intensities of sequences 2, 4, and 6 increased by 367%, 507% and 138%, respectively. Therefore, the analysis shows that I3b was the best experimental result. The specific sequence is shown in Table S1.

## 3. Materials and Methods

### 3.1. DNA Sequences and Design

All strands were modeled using Nupack and DNA sequence design minimized cross-linking between unrelated domains to avoid unwanted secondary structure formation. All strands were produced by Sangon Biotech Co., Ltd. (Shanghai, China). Unmodified DNA strands were purified by polyacrylamide gel electrophoresis (PAGE), and both RNA-modified and fluorescent-modified DNA strands were purified by high-performance liquid chromatography (HPLC) (Sangon Biotech Co., Ltd., Shanghai, China). The sequences of all strands are presented in Table S1. All DNA strands were dissolved in water and quantified using Nanodrop 2000 and the absorbance was recorded at λ = 260 nm.

### 3.2. Preparation of Buffer Conditions

The DNA strands were each diluted by 1× TAE/Mg^2+^ buffer (40 mM Tris, 20 mM acetic acid, 1 mM EDTA 2Na and 12.5 mM Mg acetate, pH 8.0). Only the strands of the hairpin structure series (Y1-5, X1, X2, I3) in all modules needed to be annealed. The annealing conditions were such that the DNA was denatured by heating at 90 °C for 10 min and then slowly cooled to 25 °C. The desired strands in each module were then mixed with the annealed hairpins and reacted at a constant temperature of 25 °C.

### 3.3. Native Polyacrylamide Gel Electrophoresis

The prepared sample was mixed with 60% glycerol 6:1, then injected into a 12% natural polyacrylamide gel, and subjected for 2 h at 4 °C at a constant voltage of 90 V in 1× TAE/Mg^2+^ buffer. Among them, the reaction system of the logic gate was 20 µL + 3 µL, and the reaction system of the logic cascade was 25 µL + 4 µL. The gel was then immersed in Stains All stain for approximately 40 min and returned to a colorless state for imaging by conducting scanning with a scanner.

### 3.4. Fluorescence Normalization

Fluorescence analysis was performed using real-time PCR (Agilent, G8830A, Agilent Technologies Inc., Palo Alto, CA, USA) equipped with a 96-well fluorescence plate reader. All fluorescence measurements were carried out at 25 °C in a reaction system of 30 µL. All fluorescence intensities were normalized, such that one normalized unit of fluorescence (n.u.) corresponded to the highest fluorescence increment of each system. All fluorescence experiments were repeated three times to ensure repeatability.

## 4. Conclusions

We propose a controllable logic circuit based on DNAzyme activity. The key point of the research is to control the activity of DNAzyme through DNA hairpin and further control the operation of logic circuit. First, a hairpin DNA containing a DNAzyme is constructed, and then the DNAzyme in the hairpin is dynamically regulated through the input strand to change the DNAzyme from an inactive state to an active state to complete a series of logical operations. On one hand, a series of DNA logic gates are constructed, including YES gate, AND gate, and INHIBIT gate. Parallel demultiplexer logic circuit and two layers of YES-YES and YES-TAND cascade circuits are constructed in a modular manner. Among them, the demultiplexer uses the same sequence as a single logic gate, which provides more powerful evidence for the improvement of reaction efficiency. On the other hand, leakage, a common phenomenon in cascade circuit, is also effectively controlled in the experiment. Two cascaded circuits were optimized by two influencing factors: concentration and DNA sequence. Through the comparative analysis of the experimental results led to the best performance. The successful operation of each module in the experiment proves the feasibility of the system to regulate the DNA logic circuit. In addition, E6 DNAzyme has the advantages of uniform sequence, easy synthesis, and good stability, indicating that this strategy is versatile in building logic circuits. The results of this experiment indicate that this regulatory strategy for DNAzyme activity also applies to more complex logic calculations. We envisage combining more methods (such as DNAzyme, restriction enzyme, G-quadruplex, and DNA origami) with DNA strand displacement, which will bring more possibilities for molecular logic calculations. This will provide more applications for future nanorobots, biosensing, and disease diagnosis.

## Supplementary Materials

The following are available online, Figure S1: The YES gate time-dependent normalized fluorescence intensity changes (ΔF/ΔFMax) at different hairpin structure, Figure S2: The YES gate time-dependent normalized fluorescence intensity changes (ΔF/ΔFMax) at different input concentrations, Figure S3: The AND gate time-dependent normalized fluorescence intensity changes (ΔF/ΔFMax) at different input concentrations, Figure S4: (A) Schematic of the TAND logic gate, (B) TAND gate logic symbol and truth table, Figure S5: Gel analysis of the TAND gate reaction using 12% PAGE, Figure S6: (A) The TAND gate time-dependent normalized fluorescence intensity change (ΔF/ΔFMax) at different inputs, (B) Comparison of time-dependent normalized fluorescence intensity changes (ΔF/ΔFMax) between AND gate and TAND gate., Figure S7: (A) The INHIBIT gate time-dependent normalized fluorescence intensity change (ΔF/ΔFMax) at different inputs, (B) Gel analysis of the INHIBIT gate reaction using 12% PAGE, Figure S8: (A) The YES-YES logic circuit time-dependent normalized fluorescence intensity change (ΔF/ΔFMax) at different concentrations, (B) Statistical analysis of system concentrations, Figure S9: Gel analysis of the YES-YES reaction using 12% PAGE, Figure S10: Nupack simulation for sequences with hairpin structures in Table S1, Figure S11: Nupack simulations for single-stranded sequences in Table S1, Figure S12: Nupack simulations for double-stranded sequences in Table S1, Table S1: DNA sequences.
