# Supplementary material for: Constructing Controllable Logic Circuits Based on DNAzyme Activity
Source: Molecules. 2019 Nov 15;24(22):4134. doi: 10.3390/molecules24224134 (PMC6891523; doi:10.3390/molecules24224134)
Supplement: Supplementary file 1 [file molecules-24-04134-s001.pdf]

Supporting Information
Constructing Controllable Logic Circuits Based on DNAzyme activity

Fengjie Yang [1], Yuan Liu [2], Bin Wang [1,*], Changjun Zhou [3,*] and Qiang Zhang [1,2,*]

[1] Key Laboratory of Advanced Design and Intelligent Computing, Dalian University, Ministry of Education, Dalian 116622, China
[2] School of Computer Science and Technology, Dalian University of Technology, Dalian 116024, China
[3] College of Computer Science and Engineering, Dalian Minzu University, Dalian, 116600, China
* Correspondence: wangbinpaper@gmail.com (B.W.); zhou-chang231@163.com(C.J.Z.); zhangq@dlut.edu.cn (Q.Z.)

Contents

1. YES gate 2
2. AND gate 4
3. TAND gate 5
4. INHIBIT gate 7
5. YES-YES cascading logic circuit 8
6. DNA sequences 10
7. References 15

1. YES gate

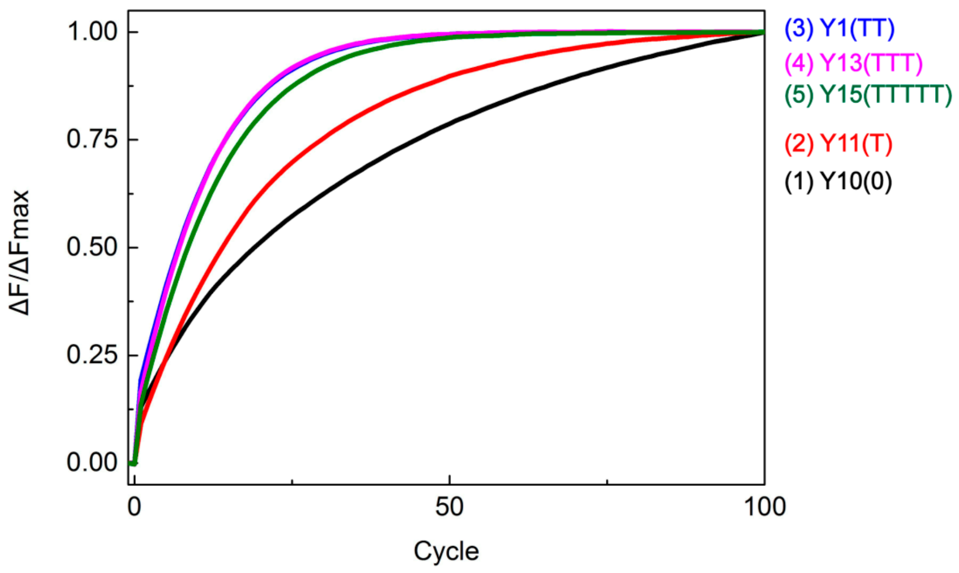

Figure S1. The YES gate time-dependent normalized fluorescence intensity changes (ΔF/ΔFMax) at different hairpin structure. Curves 1–5 reflect the change in fluorescence when the number of bases "T" at the "stem loop" junction of the hairpin is 0, 1, 2, 3, and 5, respectively. The sampling interval is 3 minutes, 100 cycles. All data represent the average of three replicates.

In order to prevent the input module from being too close to the DNAzyme, the cutting efficiency is affected. We have explored the sequence of the input strand. Next, we compared the effect of the number of bases "T" at the "stem-loop" junction of the hairpin on the reaction rate. As shown in Figure S1, the fluorescent signals of curves 1–5 all have a significant rise. However, compared to curves 3–5, the reaction rates of curve 1 and curve 2 are significantly slower. The reaction rate of curves 3–5 was almost the same, and finally we chose the hairpin structure Y1 as the experimental component.

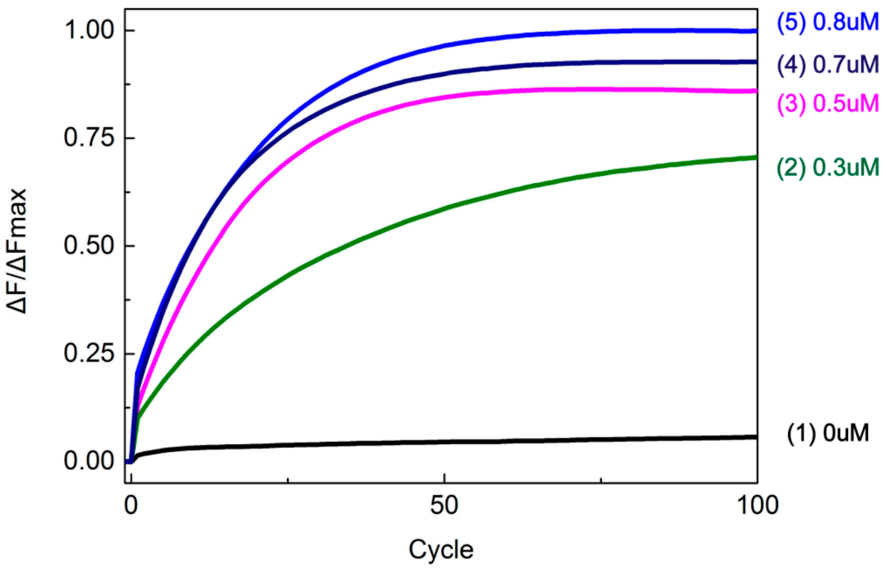

Figure S2. The YES gate time-dependent normalized fluorescence intensity changes ($\Delta F/\Delta FMax$) at different input concentrations. Curves 1─5 reflect the fluorescence changes of input strand YE-1 at 0 μM, 0.3 μM, 0.5 μM, and 0.7 μM, 0.8 μM, respectively. The other strand concentrations in the solution were maintained at 0.3 μM. The sampling interval is 3 minutes, 100 cycles. All data represent the average of three replicates.

In addition, the effect of different input strand concentrations on the "YES" gate was verified by the change in fluorescence intensity over time. The concentration of other components in the solution remained unchanged (Figure S2). Curves 2–5 show the significant increase in fluorescence intensity at concentrations of 0.3 μM, 0.5 μM, 0.7 μM and 0.8 μM. As the concentration of the input strand increases, the fluorescence intensity increases accordingly. In contrast, when there was no input, no significant increase in fluorescence signal was observed (curve 1).

2. AND gate

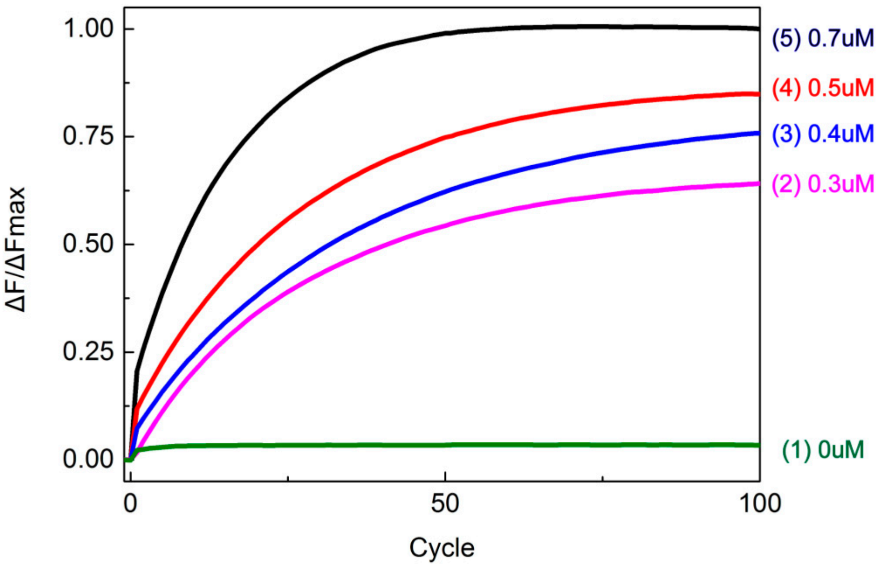

Figure S3. The AND gate time-dependent normalized fluorescence intensity changes (ΔF/ΔFMax) at different input concentrations. Curves 1–5 reflect the fluorescence changes of two input strands AN-1 and AN-2 at 0 μM, 0.3 μM, 0.4 uM, and 0.5 uM, 0.7 uM, respectively. The other strand concentrations in the solution were maintained at 0.3 μM. The sampling interval is 3 minutes, 100 cycles. All data represent the average of three replicates.

In order to better verify the input-output response, the effect of different input strand concentrations on the AND gate was designed with the concentration of other components in the solution unchanged. Real-time monitoring was performed using fluorescence (Figure S3). Curves 2–5 show the significant increase in fluorescence at concentrations of 0.3 μM, 0.4 μM, 0.5 μM, and 0.7 μM. As the concentration of the input strand increases, the reaction rate increases accordingly. In contrast, no significant increase in fluorescence signal was observed when no input was present (curve 1).

3. TAND gate

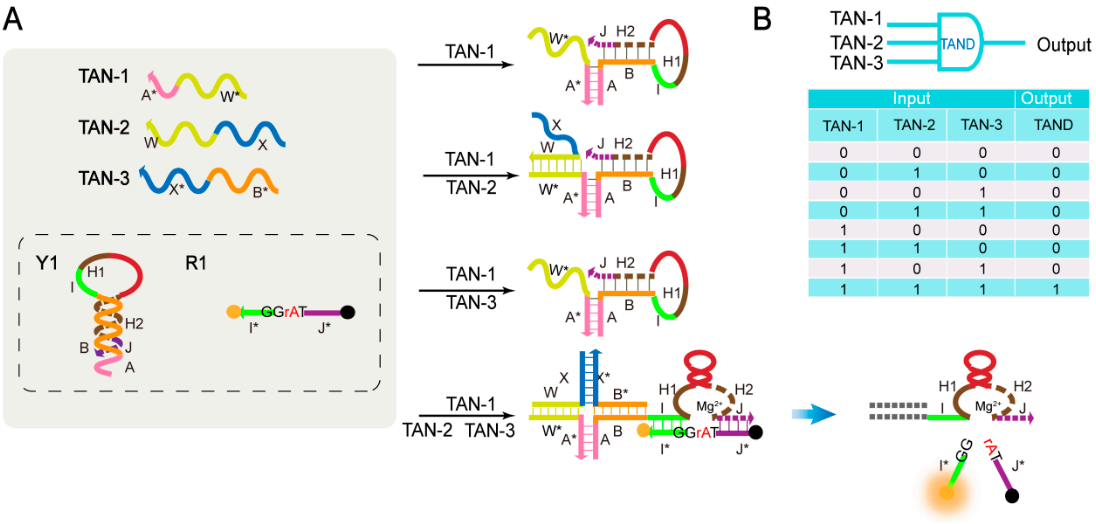

Input			Output
TAN-1	TAN-2	TAN-3	TAND
0	0	0	0
0	1	0	0
0	0	1	0
0	1	1	0
1	0	0	0
1	1	0	0
1	0	1	0
1	1	1	1

Figure S4. (A) Schematic of the TAND logic gate. Only shows the reaction with Y1. **(B)** TAND gate logic symbol and truth table. '1' means there is input, '0' means no input.

In the YES gate and the AND gate, we explored the possibility of single-input and double-input regulation of DNAzyme activity. To further prove the flexibility and variability of the system. We discussed the possibility of three-input regulation of DNAzyme activity (Figure S4 A). Similarly, TAND (Three input AND) uses the same hairpin and substrate (Y1 and R1) as the YES gate. In the TAND gate system, when TAN-1, TAN-1/TAN-2 or TAN-1/TAN-3 was added, Y1 cannot be fully opened. The structure of the DNAzyme cannot be released (and because of the weak binding strength of AN-1 and Y1, it easily falls off). Therefore, no output signal was generated. When TAN-1 is not present, no matter which of the other two input strands is present, Y1 had no exposed bases to combine, and no signal was generated. However, when TAN-1, TAN-2 and TAN-3 are simultaneously added, a stable four-way structure can be formed with Y5. When the three input strands are in close proximity, the DNAzyme in Y1 is exposed and cleaved at the recognition site (TrAGG) with ribonucleobase (rA). There is a signal output that produces a TAND gate. Therefore, three inputs are required to activate the DNAzyme, and when one or both inputs are present, the DNAzyme cannot be released and remains inactive. Figure S4 B shows a logical symbol and truth table for the TAND logic gate.

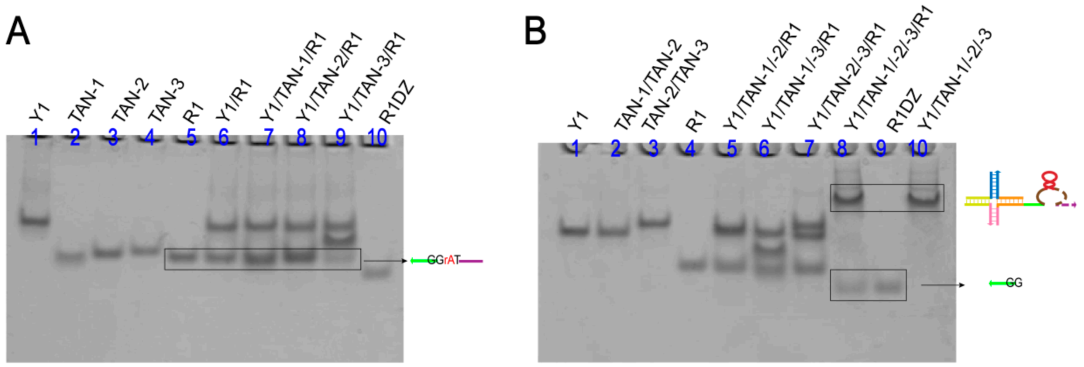

Figure S5. (A) Gel analysis of the TAND gate reaction using 12% PAGE. Lane 1: Y1; Lane 2: TAN-1; Lane 3: TAN-2; Lane 4: TAN-3; Lane 5: R1; Lane 6: Y1 and R1; Lane 7: Y1, TAN-1 and R1; Lane 8: Y1, TAN-2 and R1; Lane 9: Y1, TAN-3 and R1; Lane 10: R1DZ. **(B)** Gel analysis of the TAND gate reaction using 12% PAGE. Lane 1: Y1; Lane 2: TAN-1 and TAN-2; Lane 3: TAN-2 and TAN-3; Lane 4: R1; Lane 5: Y1,TAN-1,TAN-2 and R1; Lane 6: Y1,TAN-1,TAN-3 and R1; Lane 7: Y1, TAN-2,TAN-3 and R1; Lane 8: Y1, TAN-1,TAN-3,TAN-3 and R1; Lane 9: R1DZ; Lane 10: Y1,TAN-1,TAN-2 and TAN-3.

As shown in Figure S5 A, lanes 6-9 reflect that when no input or only one input, no new band corresponding to lane 10 is produced, no product is produced. As shown in Figure S5 B, lanes 5-7 don't produce a new band corresponding to lane 9, and no product is produced. Two new bands are generated in lane 8: one is the complex corresponding to lane 10 (TAN-1/TAN-2/TAN-3/Y1); the one corresponding to lane 9 (R1DZ). [Y1]: [TAN-1]: [TAN-2]: [TAN-3]: [R1] =1: 1.2: 1.2: 1.2: 1.5.

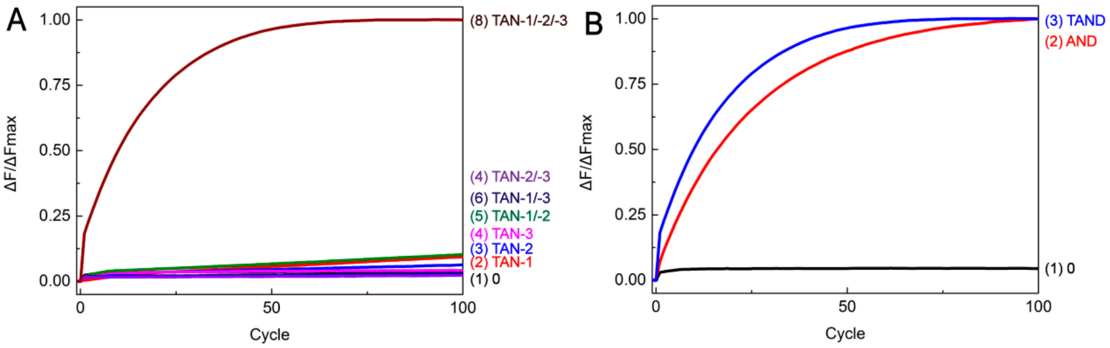

Figure S6. (A) The TAND gate time-dependent normalized fluorescence intensity change (ΔF/ΔFMax) at different inputs. The curves 1-8 reflect the change in fluorescence of the AND gate at different inputs. The time interval is 3 minutes, 100 cycles. All data represent the average of three replicates. **(B)** Comparison of time-dependent normalized fluorescence intensity changes (ΔF/ΔFMax) between AND gate and TAND gate. Curve 2 represents the change in fluorescence intensity of the AND gate. Curve 3 represents the change in fluorescence intensity of the TAND gate. The time interval is 3 minutes, 100 cycles. All data represent the average of three replicates.

Next, the TAND gate is determined by fluorescence detection. As shown in Figure S6 A, it can be observed that when the three input strands are simultaneously present (TAN-1, TAN-2 and TAN-3), the fluorescence signal is significantly enhanced (Figure S6A curve 8). Curves 1–7 reflect the absence of input, only one input or two inputs, and no significant fluorescence signal is observed. Prove the success of the TAND gate. Furthermore, in order to explore the effect of two-input and three-input on the system rate, we compared the AND gate and the TAND gate (Figure S6 B). It can be seen from the fluorescence curve that the reaction rate of the TAND gate (curve 3) is better than that of the AND gate (curve 2). It is proved that the four-way structure is more stable than the three-way structure, and this result is consistent with the principle of Multi-Helix Junction Loops.

4. INHIBIT gate

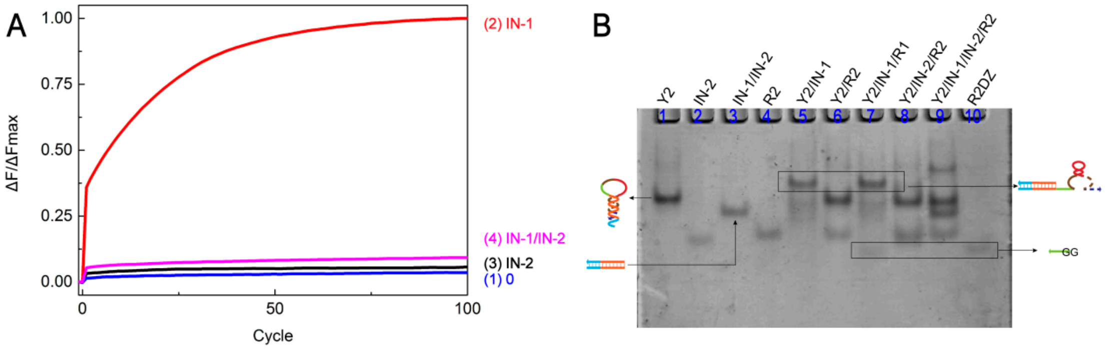

Figure S7. (A) The INHIBIT gate time-dependent normalized fluorescence intensity change (ΔF/ΔFMax) at different inputs. The curves 1-4 reflect the change in fluorescence of the INHIBIT gate at different inputs. The time interval is 3 minutes, 100 cycles. All data represent the average of three replicates. **(B)** Gel analysis of the INHIBIT gate reaction using 12% PAGE. Lane 1: Y2; Lane 2: IN-1; Lane 3: IN-1 and IN-2; Lane 4: R2; Lane 5: Y2 and IN-1; Lane 6: Y2 and R2; Lane 7: Y2, IN-1 and R2; Lane 8: Y2, IN-2 and R2; Lane 9: Y2, IN-1, IN-2 and R2; Lane 10: R2DZ.

Fluorescence detection and gel electrophoresis were used to analyze the response of the INHIBIT gate (Figure S7 A and Figure S7 B). In fluorescence detection, it was observed that the fluorescence signal was significantly enhanced in the presence of the input strand IN-1 (Figure S7 A curve 2). There was no significant increase in fluorescence intensity with no input or only o input strand IN-2 (Figure S7 A, curves 1, curves 3). Curve 4 reflects the presence of a small amount of fluorescent signal when both IN-1 and IN-2 are present. As shown in Figure S7 B, Two new bands are generated when the input strand IN-1 is added (lane 7): one is the complex corresponding to lane 5 (Y2/IN-1); the other is the strip corresponding to lane 10. [Y2]: [IN-1]: [IN-2]: [R1] =1:1.2:1.2:1.5.

5. YES-YES cascading logic circuit

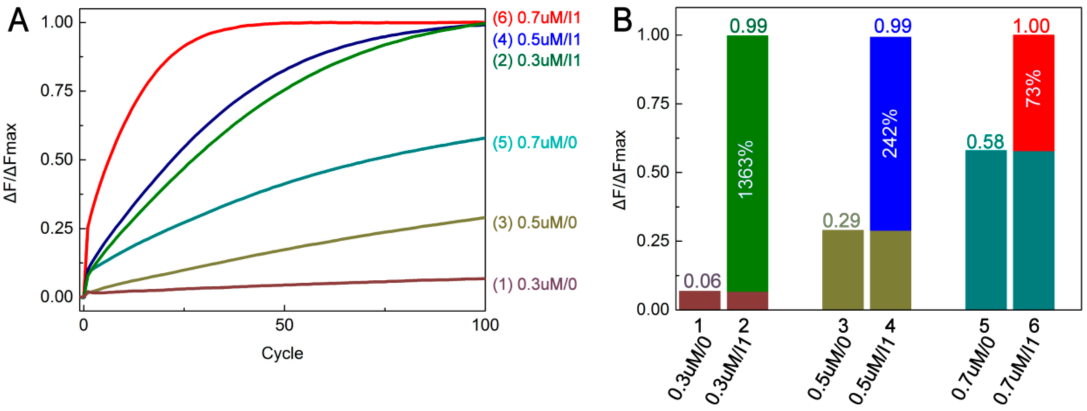

Figure S8 (A) The YES-YES logic circuit time-dependent normalized fluorescence intensity change (ΔF/ΔFMax) at different concentrations. The sampling interval is 6 minutes, 100 cycles. All data represent the average of three replicates. **(B)** Statistical analysis of system concentrations. Columns 1, 3 and 5: System analysis without I1 present, system concentrations are 0.3 μM, 0.5 μM and 0.7 μM, respectively. Columns 2, 4 and 6: Systematic analysis in the presence of I1 with system concentrations of 0.3 μM, 0.5 μM and 0.7 μM, respectively. The percentage of relative fluorescence increase ((ΔF(1) - ΔF(0))/ΔF(0)%) is indicated in columns 2, 4, and 6. The reaction time is 10 h.

We explore the effect of the concentration of the reactants on the performance of the system. The change in the fluorescence curve is reflected in Figure S8A. Among them, the substrate R2 concentration was always maintained at 0.3 μM. In the absence of I1, a different rise in the fluorescence intensity of curves 1, 3 and 5 was observed. In the case of I1, the fluorescence intensities of curves 2, 4 and 6 increased significantly. It can be analyzed that although the reaction rate is significantly increased at high concentrations, the corresponding leakage is also increased. A statistical analysis of the concentration comparison is given in Figure S8 B. Comparing the effects of the leakage in columns 1, 3 and 5, the fluorescence intensities in columns 2, 4 and 6 increased by 1363%, 242% and 73%, respectively. Comparative analysis showed that the system concentration of 0.3 μM had the best performance.

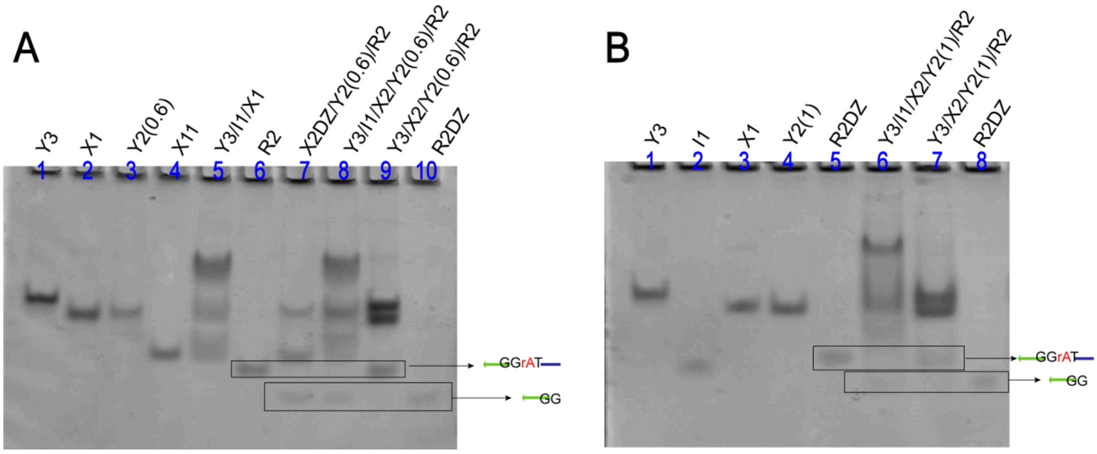

Figure S9 (A) Gel analysis of the YES-YES reaction using 12% PAGE. Lane 1: Y3; Lane 2: X1;

Lane 3: Y2; Lane 4: X1DZ; Lane 5: Y3, I1 and X1; Lane 6: R2; Lane 7: X2DZ, Y2 and R2; Lane 8: Y3, I1, X2, Y2 and R2; Lane 9: Y3, X2, Y2 and R2; Lane 10: R2DZ. **(B)** Gel analysis of the YES-YES gate reaction using 12% PAGE. Lane 1: Y3; Lane 2: I1; Lane 3: X1; Lane 4: Y2; Lane 5: R2DZ; Lane 6: Y3, I1, X2, Y2 and R2; Lane 7: Y3, X2, Y2 and R2; Lane 8: R2DZ.

The YES-YES cascade circuit was confirmed by native polyacrylamide gel electrophoresis (PAGE). As shown in Figure S9A, the lane 4 is not added to the input strand, so that R1 can be clearly seen. In the presence of I1, lane 8 creates a new band corresponding to lane 10. [Y3]: [I1]: [X2]: [Y2]: [R1] =1:1.2:1:0.6:1.5. As shown in Figure S9B, In the presence of I1, lane 6 creates a new band corresponding to lane 8. In the absence of input, the reaction was observed in lane 7 and a slight leak was observed. [Y3]: [I1]: [X2]: [Y2]: [R1] =1:1.2:1:1:1.5. Therefore, the difference between Figure S9 A and Figure S9 B is that the concentration of Y2 is different, and the leakage problem is further controlled by adjusting the concentration of Y2.

6. DNA sequences

Name	Sequences (from 5′ to 3′)	Length (n.t.)
**Y10**	TGAGCGGTAGAAACATGGGTGCTCTTCAGCGATCCGGAACGGC ACCCATGTTTCTAC	57
**Y11**	TGAGCGGTAGAAACATGGGTGTCTCTTCAGCGATCCGGAACGG CACCCATGTTTCTAC	58
**Y1**	TGAGCGGTAGAAACATGGGTGTTCTCTTCAGCGATCCGGAACG GCACCCATGTTTCTAC	59
**Y13**	TGAGCGGTAGAAACATGGGTGTTTCTCTTCAGCGATCCGGAACG GCACCCATGTTTCTAC	60
**Y15**	TGAGCGGTAGAAACATGGGTGTTTTTCTCTTCAGCGATCCGGAA CGGCACCCATGTTTCTAC	62
**YE-1a**	CCCATGTTTCTACCGCTCA	19
**YE-1b**	ACCCATGTTTCTACCGCTCA	20
**YE-1c**	CACCCATGTTTCTACCGCTCA	21
**R1**	GTAGAAT/rA/GGAAGAG	15
**R1DZ**	GGAAGAGTTTTTTTTTTTTTT	21
**AN-1a**	CGTCTGTGATCGAACGTTCGCTCA	24
**AN-2a**	CACCCATGTTTCTACTTCGTTCGATCACAGACG	33
**AN-1b**	CGTCTGTGATCGAACGTTCCGCTCA	25
**AN-2b**	CACCCATGTTTCTATTCGTTCGATCACAGACG	32
**AN-1c**	CGTCTGTGATCGAACGTTACCGCTCA	26
**AN-2c**	CACCCATGTTTCTTTCGTTCGATCACAGACG	31
**AN-1d**	CGTCTGTGATCGAACGTTTACCGCTCA	27
**AN-2d**	CACCCATGTTTCTTCGTTCGATCACAGACG	30
**AN-1e**	CGTCTGTGATCGAACGTTCTACCGCTCA	28
**AN-2e**	CACCCATGTTTTTCGTTCGATCACAGACG	29
**TAN-1**	CGTGAGAGTCAGAACGTTACCGCTCA	26
**TAN-2**	GAGTAGCATCAGTGAGTTCGTTCTGACTCTCACG	34
**TAN-3**	CACCCATGTTTCTTTCTCACTGATGCTACTC	31
**Y2**	CTAGCCTGTGCTACATGGGTGTTGACCTCAGCGATCCGGAACGG CACCCATGTAGCACA	59
**IN-1**	CACCCATGTAGCACAGGCTAGGCC	24
**IN-2**	GGCCTAGCCTGTGCTACATGGGTG	24

**R2**	TGTGCTT/rA/GGAGGTC	15
**R2DZ**	GGAGGTCTTTTTTTT	15
**DE-1**	CACCCATGTTTCTTTCACCCATGTAGCACAGGCTAGCGG	39
**DE-2**	CCGCTAGCCTGTGCTACATGGGTGTTACCGCTCA	34
**Y3**	CTGCTCGTCTAACCACATGGGTGTTCAGTACAGCGATCCGGAAC GGCACCCATGTGGTTAGACTC	65
**I1**	CACCCATGTGGTTAGACGAGCAG	23
**X1**	CTAGCCTGGAGTCTAACCT/rA/GGTACTGTTTTTCACCCATGTAG CACAGGCTAG	53
**X11**	GGTACTGTTTTTCACCCATGTAGCACAGGCTAG	33
**Y2a**	AGCCTGTGCTACATGGGTGTTGACCTCAGCGATCCGGAACGGCA CCCATGTAGCACA	57
**Y2b**	TAGCCTGTGCTACATGGGTGTTGACCTCAGCGATCCGGAACGGC ACCCATGTAGCACA	58
**Y4**	CTCGACATCCAGCATACATGGGTGTTCTCTTCAGCGATCCGGAA CGGCACCCATGTATGCTGGATC	66
**I2**	CACCCATGTATGCTGGATGTCGAG	24
**X2**	GCGTCAACGATCCAGCATT/rA/GGAAGAGGACAGTTGACGCAC CTGGTC	47
**X21**	GGAAGAGGACAGTTGACGCACCTGGTC	27
**Y5**	GACCAGGTAGAAACATGGGTGTTCTCTTCAGCGATCCGGAACG GCACCCATGTTTCTAC	59
**I3a**	GGTCAGTAGTCGTTGCGTCAACTGTC	26
**I3b**	GGTCAGTAGTCGTTGCGTCAACTGTCTTTGACAGTT	36
**I3c**	GGTCAGTAGTCGTTGCGTCAACTGTCTTTGACAGTTG	37
**I4**	CACCCATGTTTCTTTCGACTACTGACC	27

Table S1. DNA sequences.

All of the sequences used in this work were designed using Nupack [1-3].

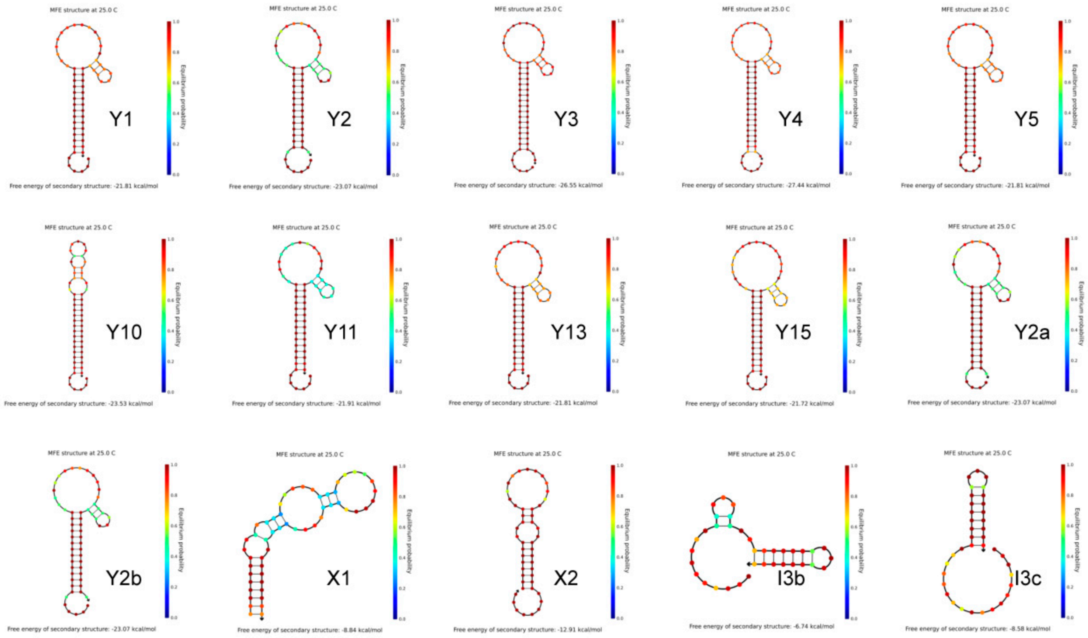

Figure S10. Nupack simulation for sequences with hairpin structures in Table S1.

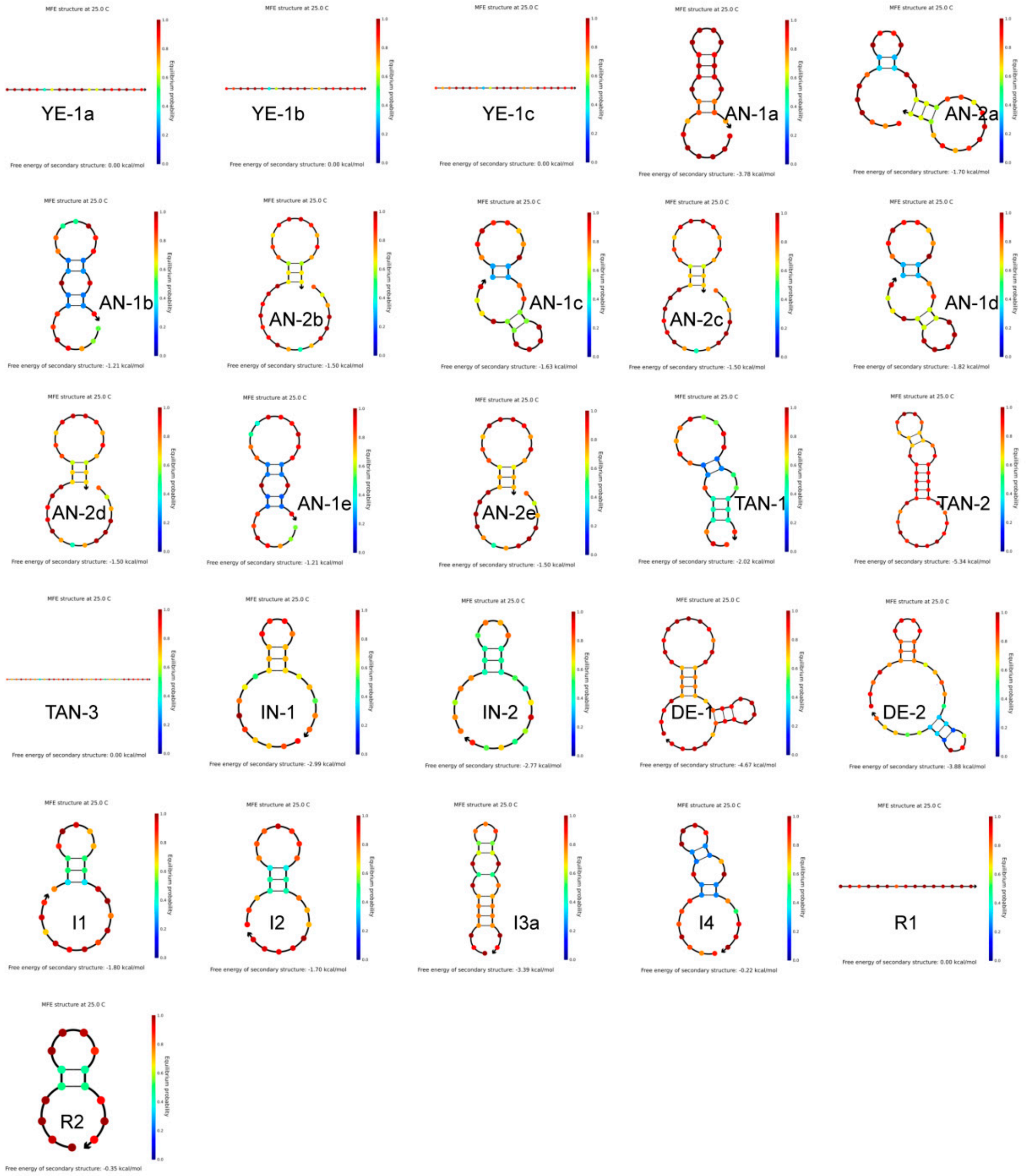

Figure S11. Nupack simulations for single-stranded sequences in Table S1.

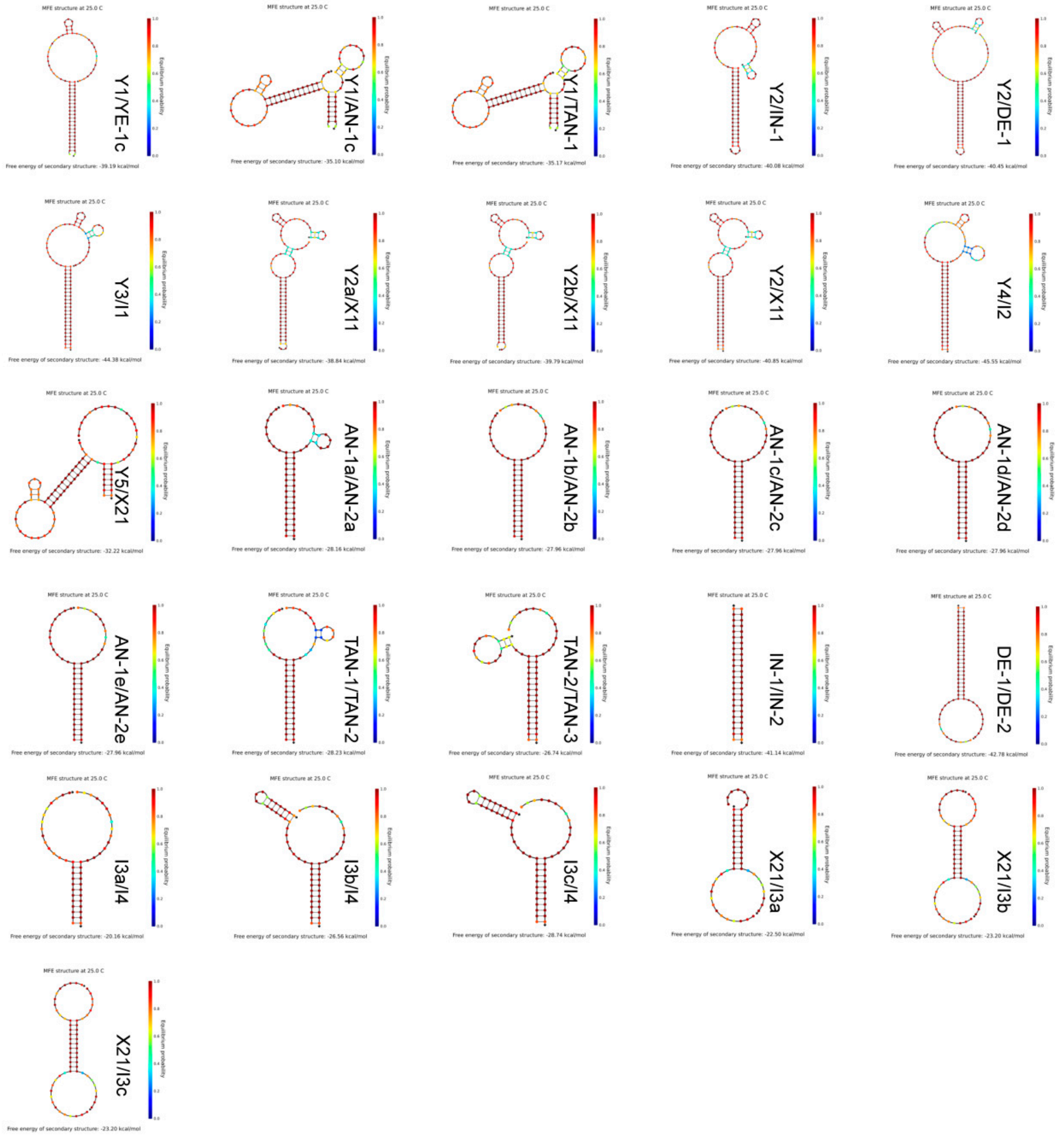

Figure S12. Nupack simulations for double-stranded sequences in Table S1.

7. References

1. Wolfe, B. R.; Porubsky, N. J.; Zadeh, J. N.; Dirks, R. M.; Pierce, N. A. Constrained multistate sequence design for nucleic acid reaction pathway engineering. *J. Am. Chem. Soc.* **2017,** *139*, 3134–3144.
2. Zadeh, J. N.; Steenberg, C. D.; Bois, J. S.; Wolfe, B. R.; Pierce, M. B.; Khan, A. R.; Dirks, R. M.; Pierce, N. A. NUPACK: analysis and design of nucleic acid systems. *J. Comput. Chem.* **2011,** *32*, 170–173.
3. Wolfe, B. R.; Pierce, N. A. Sequence design for a test tube of interacting nucleic acid strands. *Acs Synth. Biol.* **2014,** *4*, 1086–1100.
